# The effectiveness of repeated sucrose for procedural pain in neonates in a longitudinal observational study

**DOI:** 10.3389/fpain.2023.1110502

**Published:** 2023-02-07

**Authors:** Mariana Bueno, Marilyn Ballantyne, Marsha Campbell-Yeo, Carole A. Estabrooks, Sharyn Gibbins, Denise Harrison, Carol McNair, Shirine Riahi, Janet Squires, Anne Synnes, Anna Taddio, Charles Victor, Janet Yamada, Bonnie Stevens

**Affiliations:** ^1^ Child Health Evaluative Sciences, The Hospital for Sick Children, Toronto, ON, Canada; ^2^ Lawrence S. Bloomberg Faculty of Nursing, University of Toronto, Toronto, ON, Canada; ^3^ Bloorview Research Institute, Holland Bloorview Kids Rehabilitation Hospital, Toronto, ON, Canada; ^4^ Departments of Psychology & Neuroscience and Pediatrics, Faculty of Health, School of Nursing, Dalhousie University, Halifax, NS, Canada; ^5^ Centre for Pediatric Pain Research, IWK Health Centre, Halifax, NS, Canada; ^6^ Faculty of Nursing, University of Alberta, Edmonton, AB, Canada; ^7^ Trillium Health Partners, Mississauga, ON, Canada; ^8^ Department of Nursing, The University of Melbourne, Melbourne, VIC, Australia; ^9^ Clinical Sciences and Nursing, Murdoch Children's Research Institute and Royal Children's Hospital, Melbourne, VIC, Australia; ^10^ Faculty of Health Sciences, School of Nursing, University of Ottawa, Ottawa, ON, Canada; ^11^ Neonatology, The Hospital for Sick Children, Toronto, ON, Canada; ^12^ Pediatrics, University of British Columbia, Vancouver, BC, Canada; ^13^ Leslie Dan Faculty of Pharmacy, University of Toronto, Toronto, ON, Canada; ^14^ The Institute of Health Policy, Management and Evaluation, University of Toronto, Toronto, ON, Canada; ^15^ Daphne Cockwell School of Nursing, Toronto Metropolitan University, Toronto, ON, Canada

**Keywords:** neonate, pain, procedural pain, pain assessment, sucrose, effectiveness

## Abstract

**Goal:**

To determine the analgesic effectiveness of repeated sucrose administration for skin-breaking (SB) procedures over the Neonatal Intensive Care Unit (NICU) hospitalization of preterm infants.

**Methods:**

Longitudinal observational study, conducted in four level III Canadian NICUs. Eligible infants were <32 weeks gestational age at birth, and <10 days of life at enrollment. Infants received 24% sucrose (0.12 ml) prior to all painful procedures. The Premature Infant Pain Profile – Revised (PIPP-R) was used at 30 and 60 seconds after a medically-required SB procedure as soon as possible after enrollment and weekly up to three additional times for scheduled procedures.

**Results:**

172 infants (57.3% male, gestational age 28.35 (±2.31) weeks) were included. The mean 30 s PIPP-R scores were 6.11 (±3.68), 5.76 (±3.41), 6.48 (±3.67), and 6.81 (±3.69) respectively; there were no statistically significant interactions of study site by time (*p* = 0.31) or over time (*p* = 0.15). At 60 s, mean PIPP-R scores were 6.05 (±4.09), 5.74 (±3.67), 6.19 (±3.7), and 5.99 (±3.76) respectively; there were no study site by time interactions (*p* = 0.14) or differences over time (*p* = 0.52). There was a statistically significant site difference in the effectiveness of sucrose at 30 and 60 seconds (*p* < 0.01).

**Conclusions:**

Consistently low PIPP-R scores following a skin-breaking procedure indicated that the analgesic effectiveness of the minimal dose of sucrose was sustained over time in the NICU. Further research is required to determine the optimal combination of sucrose and other pain management strategies to improve clinical practice and the impact of consistent use of repeated use of sucrose on neurodevelopment.

## Introduction

Sucrose has been extensively investigated as an intervention to reduce procedural pain in term and preterm infants since the early 1990s. Systematic reviews and meta-analyses updated regularly have consistently demonstrated the analgesic effectiveness of sucrose administered during single skin-breaking (SB) procedures (1–3). However, the effectiveness and safety of repeated sucrose administration to hospitalized preterm neonates during their full NICU stay has been identified as a significant knowledge gap.

Sick and preterm neonates undergo numerous painful procedures per day during hospitalization in the NICU (4–6) and require multiple interventions for pain relief. Gao et al. (7) conducted a systematic review to assess the efficacy and safety of repeated administration of sucrose for procedural pain in neonates. Four of eight studies identified reported pain intensity scores over repeated painful procedures (such as heel lancing, venipuncture, and intramuscular injection) in term and preterm infants; their results indicated that 0.1 ml to 2.0 ml of 20%–25% of sucrose was effective in reducing scores measured by a validated neonatal pain tool. Meta-analysis were not conducted due to large variability across research methodologies and pain intensity outcomes adopted in the included studies. Given the widely reported dosing range across trials, Stevens et al. compared the effects of three doses of 24% sucrose (0.1 ml; 0.5 ml; 1.0 ml) associated with heel lances in preterm and term neonates (8). There were no significant differences on pain intensity scores measured using the Premature Infant Pain Profile – Revised - PIPP-R (9) among the treatment groups; thus, the authors suggested that the minimally effective dose of 24% sucrose for a single heel lance was 0.1 ml. However, the effectiveness of repeatedly administering this dose for multiple painful procedures over the full NICU hospital stay has yet to be explored.

We hypothesized that, in preterm neonates undergoing painful procedures during hospitalization in the NICU, repeated sucrose administration of the minimally effective sucrose dose (0.1 ml of 24% sucrose solution) for medically-required heel lance, would be consistently effective in decreasing pain intensity over time.

## Methods

### Design and settings

A longitudinal observational study was conducted in four level III university-affiliated NICUs in central and eastern Canada.

### Participants

Infants, hospitalized in the NICU who were (i) <32 weeks gestational age (GA) at birth, (ii) expected to have medically-required heel lances, and (iii) <10 days of life (DOL) at the time of study enrollment were eligible for study inclusion. No lower gestational age limit was imposed. Infants receiving analgesics were included, as this does not preclude sucrose use during SB procedures. Infants were excluded if they had contraindications for sucrose administration.

### Procedures for data collection

Following approval by the Research Ethics Boards (REB) at all participating sites, parents of eligible infants were approached by a research nurse, who explained the study and obtained written consent.

From study enrollment to discharge from the NICU, bedside nurses were asked to administer sucrose with each SB or potentially SB painful procedure. Infants received a single dose of 0.12 ml (i.e., three drops) of 24% sucrose onto the anterior surface of the tongue, 2 min prior to every painful procedure, over a period of no more than 1 min. A pacifier was offered following sucrose administration, if the infant was able to hold the pacifier securely to induce non-nutritive sucking (NNS). The sucrose study dose (0.12 ml) was repeated as a rescue dose if the procedure continued over one minute or if the infant showed signs of moderate to severe distress (e.g., grimacing, crying, changes in vital signs). No maximum procedural, daily, or cumulative sucrose dose limit was established. Bedside nurses administered and documented sucrose administration on the infant's medical record.

Data were collected by the site research nurse and managed using REDCap electronic data capture tools (10, 11) and hosted at the institution of the principal investigator. Data monitoring and logistic checks were performed throughout data collection.

### Outcome

The primary outcome, pain intensity, was measured using the PIPP-R pain assessment tool. The original PIPP was developed by Stevens in 1996 (12) and updated as the PIPP-R with minor revisions, primarily to scoring in 2014 (9, 13). The PIPP-R includes 2 physiological (heart rate, oxygen saturation), 3 behavioral (brow bulge, eye squeeze, nasolabial furrow), and 2 contextual (gestation age [GA], behavioral state [BS]) variables known to modify pain responses (14). Each variable is scored on a four-point scale (0–3) for a potential of 21 points for pre-term infants; GA and BS are reverse-scored to account for developmental and sleep state contextual differences in preterm and term infants' abilities to respond to pain (12). In the PIPP-R, ordering and scoring of the 2 contextual variables, GA and BS, have been changed to ensure that baseline characteristics prior to the painful event do not artificially inflate scores (9, 13).

PIPP-R scores were measured with a SB procedure (i.e., heel lance) as soon as possible upon study enrolment (T1) and then weekly for medically-required heel lance procedures up to three additional times (T2-T4). PIPP-R scores were assessed 30 and 60 seconds following the invasive phase (e.g., needle insertion) of the procedure. PIPP-R scores were measured in real time by trained research nurses who achieved an inter-rater reliability >0.8, using extensive training methods established by the authors and implemented in previous research (13, 15, 16).

### Sample size calculation

We estimated that approximately 40 infants per site would adequately represent the population of interest and meet the study goals in this longitudinal study design. We also attempted to oversample by 10% to account for loss to follow-up. In total, 172 infants were recruited for the study.

### Data analysis

Descriptive statistics were used to describe the effectiveness of sucrose across time and between sites. Mixed effects linear models were conducted to examine the association between PIPP-R scores and sucrose administration over time.

## Results

Of the 172 infants enrolled in the study,153 had at least 1 pain assessment; a total of 418 pain assessments were recorded for the full sample from T1 and T4. The vast majority of painful procedures assessed were heel lances (94.2%), considered to be of moderate pain intensity using the pain intensity rating scale by Laudiano-Dray et al. (17); when the infant did not require a heel lance, another SB procedure of similar pain intensity that the infant was undergoing such as intravenous start was substituted for study purposes (5.8%).

Infant characteristics are described in Table 1, overall and by study site. Detailed data on the nature and frequency of painful procedures, sucrose administration, pain-reducing interventions and adverse events throughout the NICU stay are reported elsewhere (18).

**Table 1:** Infant demographics, overall and by site (*n* = 172).

Characteristics		Overall	Site 1	Site 2	Site 3	Site 4	*p*-value
Sex*	F	73 (42.7)	27 (52.9)	34 (45.3)	6 (25.0)	6 (28.6)	0.086
	M	98 (57.3)	24 (47.1)	52 (54.7)	18 (75.0)	15 (71.4)	
GA at birth in weeks**	28.35 (2.31)	27.95 (2.31)	28.95 (2.24)	26.74 (1.88)	29.01 (1.99)	<0.00
BW in grams**	1096.54 (382.02)	1048.63 (304.91)	1181.77 (336.26)	806.59 (374.45)	1239.87 (522.40)	0.005

* Data reported as *N*(%).

** Data reported as Mean (Standard Deviation - SD).

PIPP-R scores across the four data collection points (T1–4) during the infant's hospitalization indicated that there was no significant difference in pain intensity over time by site (time × site interaction) or by time. There was a significant difference by site (Tables 2, 3).

**Table 2:** PIPP-R scores at 30 seconds after the procedure over time.

30 sec	Overall		Site 1		Site 2		Site 3		Site 4	
Time	*N*	Mean (SD)	*N*	Mean (SD)	*N*	Mean (SD)	*N*	Mean (SD)	*N*	Mean (SD)
T1	136	6.11 (3.68)	25	8.0 (4.05)	73	5.04 (2.84)	24	7.25 (4.76)	14	6.36 (3.13)
T2	114	5.76 (3.41)	20	7.20 (4.15)	62	5.10 (2.30)	21	6.62 (4.75)	11	5.27 (3.52)
T3	93	6.48 (3.67)	11	9.45 (4.70)	53	5.66 (2.46)	21	6.71 (4.69)	8	7.25 (4.23)
T4	75	6.81 (3.69)	14	6.36 (4.60)	41	6.15 (3.25)	14	8.93 (4.03)	6	7.50 (1.64)
*p*-value for differences over time	*F* = 1.80 *p* = 0.15	*F* = 1.20 *p* = 0.32	*F* = 1.90 *p* = 0.13	*F* = 0.84 *p* = 0.48	*F* = 0.81 *p* = 0.50

*p*-value for differences by site: *F* = 10.21; *p* < 0.001.

*p*-value for site by time differences: *F* = 1.17; *p* = 0.31.

**Table 3:** PIPP-R scores at 60 seconds after the procedure over time.

60 sec	Overall		Site 1		Site 2		Site 3		Site 4	
Time	*N*	Mean (SD)	*N*	Mean (SD)	*N*	Mean (SD)	*N*	Mean (SD)	*N*	Mean (SD)
T1	136	6.05 (4.09)	25	9.32 (4.29)	73	4.82 (3.13)	24	6.83 (4.86)	14	5.29 (3.65)
T2	114	5.74 (3.67)	20	7.75 (5.00)	62	5.05 (2.46)	21	6.05 (4.73)	11	5.36 (3.35)
T3	93	6.19 (3.70)	11	10.73 (4.15)	53	5.36 (2.40)	21	6.10 (4.38)	8	5.75 (4.50)
T4	75	5.99 (3.76)	14	6.21 (5.16)	41	5.32 (3.01)	14	7.50 (4.15)	6	6.50 (3.51)
*p*-value for differences over time	*F* = 0.76 *p* = 0.52	*F* = 2.39 *p* = 0.077	*F* = 0.49 *p* = 0.69	*F* = 0.38 *p* = 0.76	*F* = 0.17 *p* = 0.92

*p*-value for differences by site: *F* = 15.10; *p* < 0.001.

*p*-value for site by time differences: *F* = 1.53; *p* = 0.14.

Pain intensity scores on the PIPP-R at both 30 and 60 seconds were generally low (i.e., on average 6 or less out of a possible 21), indicating minimal pain on the PIPP-R and the continued effectiveness of sucrose for painful procedures over time.

## Discussion

Sucrose effectiveness, supported by a lack of significant interaction (i.e., site by time) and the main of time indicated that sucrose was effective in minimizing procedural pain in preterm infants during their NICU stay, regardless of the number of previous painful procedures or the cumulative volume of sucrose administered. This intervention effectiveness was observed through consistently low mean pain intensity scores on the PIPP-R at 30 and 60 seconds after the most invasive phase (i.e., lance) of the SB procedures, across several data collection points. Both (a) pain reactivity (pain response at the time of the procedure up to 30 seconds after the procedure) that reflects the body's immediate reaction to a painful stimulus and (b) pain regulation (pain response at 60 seconds after the painful stimulus representing the individual's recovering from the painful insult (19) are important to consider. Heel lances are considered to be moderately painful (17), which would be translated by PIPP-R scores between 7 and 12. However, the vast majority of mean PIPP-R scores at 60 seconds after the procedure indicate mild pain (scores ≤6) during recovery confirming the effectiveness of sucrose on pain relief.

There was a significant difference in the effectiveness of sucrose at both 30 and 60 seconds across sites. Two of the four sites indicated either higher pain intensity scores on average (Site 1) or lower pain intensity scores on average (Site 2). We hypothesized that the differences between these two sites may be explained by their NICU sucrose administration policy. In one site (Site 2) there was strict adherence to the study protocol where sucrose was administered 2 min prior to the painful procedures for all painful procedures and lower PIPP-R average scores. In the second site (Site 1) nurses noted that they administered the sucrose intermittently over the first minute vs. the bolus administration at one point in time as per the protocol and had higher average PIPP-R scores. Given the small volume of sucrose in total, we can speculate that this intermittent administration may have diluted the analgesic effectiveness; however, this was not a specific research question in this study. Sucrose administration modalities in very preterm infants need to be further investigated as well as whether intermittent vs. bolus administration impacts effectiveness when using such small doses.

Although sucrose has been extensively investigated and evidence synthesized for single painful procedures (1–3), there is much less evidence for the effectiveness of sucrose being administered repeatedly during hospitalization for medically-required diagnosis and treatment.

In a randomised clinical trial, Stevens et al. allocated 66 preterm infants (mean GA 27.13 to 27.67 weeks) to one of three groups: standard care (positioning and swaddling), sterile water plus non-nutritive sucking (NNS) or 24% sucrose (0.1 ml) plus NNS prior to all painful procedures in the NICU during the first 28 days of life (20). The PIPP was used to assess pain intensity during routine heel lances at 7, 14, 21, and 28 days of life. The combination of sucrose and NNS resulted in lower pain scores over time, compared to NNS or standard care. Similarly, in a longitudinal cohort study by Harrison et al., 33% sucrose (0.05 to 0.25 ml) was administered prior to all heel lancing procedures performed in 55 infants during their hospitalization; 443 pain assessments were conducted and results indicated low behavioral (crying time and grimacing) responses and minimal changes in physiological indicators to repeated lancing procedures over time (21).

Gao et al. (22) investigated the effects of sucrose, alone or combined with NNS on pain intensity for three non-consecutive heel lances in preterm infants (mean GA 31.7 [±0.9] weeks). The administration of 0.2 ml of 20% sucrose combined with NNS resulted in significantly lower PIPP scores; 20% sucrose alone (0.2 ml) and NNS alone were more effective than standard care in reducing pain for repeated heel lancing.

Campbell-Yeo et al. (23) compared the effectiveness of 24% sucrose, skin-to-skin care, and 24% sucrose combined with skin-to-skin care administered prior to all painful procedures during the NICU stay of preterm infants. Sucrose volumes administered followed the institution's sucrose administration protocol and ranged from 0.4 to 1.0 ml, depending on the infant's weight. There was sustained analgesic effect for all of the interventions over time, measured using the PIPP throughout three non-consecutive, medically indicated heel lances distributed across hospitalization.

The results of this study, in conjunction with previously published research, provides evidence of the effectiveness of sucrose administration over time in neonates. These results are important in light of evidence suggesting that repeated procedural pain might contribute to altered neurological brain development (24–28). However, there is limited evidence on neurodevelopmental outcomes of preterm infants repeatedly exposed to sucrose in the early stages of brain development (29).

It is important to acknowledge that although pain is decreased with repeated sucrose administration, it is not eliminated but rated as minor according to the scoring algorithm on the PIPP. Therefore, sucrose should be administered cautiously or in conjunction with other evidence-based non-pharmacological interventions whenever possible (e.g., NNS, facilitated tucking, skin-to-skin care, among others (30, 31).

## Limitations of the study

Limitations were mainly related to assessment of pain intensity, fidelity of delivering the intervention and loss to follow-up in longitudinal designs. Pain assessment is challenging, particularly in very preterm neonates (32, 33). Performing bedside pain assessment in real time is even more challenging. Although composite measures, such as the PIPP-R, are the most recommended tools for pain assessment in neonates and infants, it is difficult to capture the complexity of pain experience in this particular population in real time. There is growing interest in the infants' brain activity in pain mechanisms (31, 34) and further investigation may provide a more comprehensive approach on understanding and managing neonatal and infant pain expression and experience. Continuous monitoring of the infant, rather than episodic observations, may also prove more fruitful in capturing the infant's pain experience.

Challenges in implementing the intervention consistently across the sites as intended (fidelity) may have occurred due to differences in sucrose administration practice, influenced by local policies and the research protocol. Research nurses received training related to the study administration of sucrose but training for the large number of bedside nurses working on the units was reliant on the time and availability of the research nurse and may have differed from local practices. This might have resulted in sucrose being administered differently across sites. In addition, pain assessment following a painful procedure with no sucrose administration at baseline was not performed due to ethical considerations that recommend studies not be conducted without acceptable analgesia to all babies enrolled, if such pain relief exists (35, 36).

Finally, there was a significant loss to follow-up resulting in not all neonates being observed during medically required blood tests at the four data collection points of the study. This was a result of the infants' discharge or transfer from the NICU, unavailability of the research nurse for assessing pain, or no requirement for SB procedures. These challenges are consistent with long-term observation studies.

## Conclusion

Consistently low PIPP-R scores were reported for SB procedures over the NICU stay, confirming sustained analgesic effectiveness of a minimal amount sucrose for repeated painful procedures in the NICU. However, mild pain was still assessed. We used the established minimally effective dose of sucrose for neonates so that infants would receive the smallest cumulative amount of sucrose possible throughout the NICU stay. Ideal analgesia for needle-related procedures in neonates might be achieved with the cautious administration of sucrose in partnership with other pain relief strategies, such as NNS, breastfeeding, facilitated tucking and skin-to-skin care. Further research is required to determine the optimal mix of strategies to improve clinical practice and achieve optimal infant outcomes. Additionally, research on the impact of repeated sucrose on neurodevelopment is also warranted.

## Data Availability

The original contributions presented in the study are included in the article/Supplementary Materials, further inquiries can be directed to the corresponding author/s.
